# Real-time three-dimensional fluoroscopy-navigated percutaneous pelvic screw placement for fragility fractures of the pelvis in the hybrid operating room

**DOI:** 10.1186/s12891-022-06026-w

**Published:** 2022-12-03

**Authors:** Mika Takaesu, Satoshi Nakasone, Yoshihide Miyata, Kotaro Nishida

**Affiliations:** 1 Department of Orthopaedic Surgery, Chubu Tokushukai Hospital, 801 Higa, Kitanakagusuku, Nakagami-gun, Okinawa, 901-2393 Japan; 2 Department of Orthopaedic Surgery, Graduate School of Medicine, University of the Ryukyus, 207 Aza-Uehara, Nishihara, Nakagami-gun, Okinawa, 903-0215 Japan

**Keywords:** Fragility fractures of the pelvis, Percutaneous screw, Hybrid operating room, 3D fluoroscopic navigation, C-arm cone-beam CT, Needle guidance

## Abstract

**Background:**

The prognosis of conservative treatment for fragility fracture of the pelvis (FFP) in the older patients remains poor. Percutaneous pelvic screw placement (PPSP), which aids in the treatment of FFP, can be challenging to perform using fluoroscopy alone because of the proximity of blood vessels and neuroforamina. Hence, this study aimed to investigate the accuracy and clinical outcomes of PPSP using real-time 3D fluoroscopic navigation for FFP in the hybrid operating room.

**Methods:**

This study included 41 patients with FFP who underwent PPSP in a hybrid operating room between April 2016 and December 2020. Intraoperative C-arm cone-beam CT was performed under general anesthesia. Guidewire trajectory was planned using a needle guidance system. The guidewire was inserted along the overlaid trajectory using 3D fluoroscopic navigation, and a 6.5 mm cannulated cancellous screw (CCS) was placed. The clinical outcomes and accuracy of the screw placement were then investigated.

**Results:**

A total of 121 screws were placed. The mean operative time was 84 ± 38.7 minutes, and the mean blood loss was 7.6 ± 3.8 g. The mean time to wheelchair transfer was 2 days postoperatively. Pain was relieved in 35 patients. Gait ability from preoperative and latest follow-up after surgery was maintained in 30 (73%) patients. All 41 patients achieved bone union. Of the 121 screws, 119 were grade 0 with no misplacement; only 2 patients had grade 1 perforations.

**Conclusion:**

PPSP using real-time 3D fluoroscopic navigation in a hybrid operating room was accurate and useful for early mobilization and pain relief among older patients with FFP with an already-installed needle biopsy application.

**Supplementary Information:**

The online version contains supplementary material available at 10.1186/s12891-022-06026-w.

## Background

The rate of fragility fracture of the pelvis (FFP) has increased due to the increase in the aging population. FFP result from low-energy trauma, such as falling from a standing position and can even occur spontaneously in patients with severe osteoporosis. The one-year mortality after pelvic fractures in older people ranges from 10 to 28.3% [1–4]. The main causes of death after fragility fractures include cardiac disease, malignancy, and respiratory disease [4]. Most FFPs are minimally displaced and do not require surgery [5]. However, the outcome of conservative therapy is poor in older patients and ambulation with full weight bearing can take a long time and result in a loss of social and physical independence and autonomy [2]. A decline in activities of daily living should be prevented in the treatment of FFP in older patients.

Minimally invasive percutaneous pelvic screw placement (PPSP), such as iliosacral (IS) screws, transiliac transsacral (TITS) screws, antegrade or retrograde pubic screws, and iliac wing screws, are useful for pain relief and early ambulation of patients with FFP with minimal dislocation. However, the screw placement in FFP is close to the neuroforamina and blood vessels and screw placement under fluoroscopy is difficult because of the narrow safety zone. Around 5–60% of FFP patients were reported to have poor screw placement accuracy and 1–23% had neurological disorders [6–11]. Although computed tomography (CT)-based navigation with intraoperative CT imaging has been reported to improve the accuracy of screw placement by navigating the position and direction, misplacement has been reported in 19–31% patients [10, 11]. Even a slight error in the registration in CT-based navigation could lead to a misplacement of the screw due to a significantly narrow safety zone.

The number of hybrid operating rooms has been increasing in recent years, and the usefulness of hybrid operating rooms has been reported not only in cardiovascular surgery but also in spine and trauma surgeries [12–14]. In the hybrid operating room, a stationary X-ray system has a maximum field of view of approximately 48 cm and produces high-quality fluoroscopic images due to the high-resolution flat-panel detector. In addition, the C-arm recognizes the radiolucent operating table and enables intraoperative real-time C-arm cone-beam CT (CBCT) imaging. This function allows the use of real-time three-dimensional (3D) fluoroscopic navigation with the ability to overlay intraoperative 3D-CT image images with real-time fluoroscopic images without registration. We hypothesized that the use of PPSP in the hybrid operating room has the potential to provide safe and minimally invasive surgery as the screws can be placed using navigation via real-time 3D fluoroscopic images overlayed on intraoperative 2D images. To the best of our knowledge, there have been no previous reports of this type of navigation, and this technique is simple, easy, and commonly used. The present study investigated the accuracy and clinical outcomes of PPSP using real-time 3D fluoroscopic navigation for FFP in the hybrid operating room.

## Methods

### Patients

The institutional review board approved this single-center, retrospective study. A total of 41 patients who underwent PPSP for FFP in a hybrid operating room from April 2016 to May 2021 were included in the study. There were seven male and 34 female patients and the mean age was 79 (46–98) years. The most frequently observed mechanisms of injury included falling from a standing position (*n* = 36), carrying a heavy object (*n* = 1), and injuries that occurred without a trigger (*n* = 4). The mean observation period was 27 ± 14 months (Table 1). Indications for surgery were patients who were able to walk before the injury and who had severe pain. Patients were excluded as they did not wish to undergo surgery, did not have spontaneous pain, were in a poor general condition and were at high risk of surgery, or the family did not consent to the patient undergoing surgery due to old age. FPP was evaluated according to Rommens classifications [5] (Table 2).

**Table 1:** Patient demographics

Characteristics	Value
Number of patients (n)	41
Sex	
Male (n)	7
Female (n)	34
Mean age (years)	79 (46–98)
Mean BMI (kg/m^2^)	22 (18–27)
Mechanism of injury (n)	
falling from the standing position	36
carrying a heavy object	1
occurred without a trigger	4
Mean follow-up period (months)	27 (12–53)

**Table 2:** FFP classification

Rommens classification [5]	
IIa	1
IIb	17
IIc	14
IVa	2
IVb	6
IVc	1

PPSP was determined as follows. TITS screws were indicated in sacral fractures when a safe corridor > 8 mm was identified in the sacral axial view. Otherwise, a unilateral IS screwing was planned. For pubic screws, antegrade pubic screws were planned when the fracture was located in the proximal middle of the superior pubic ramus and retrograde pubic screws were planned when the fracture was located in the distal middle of the superior pubic ramus.

- Surgery

**Fig. 1:**
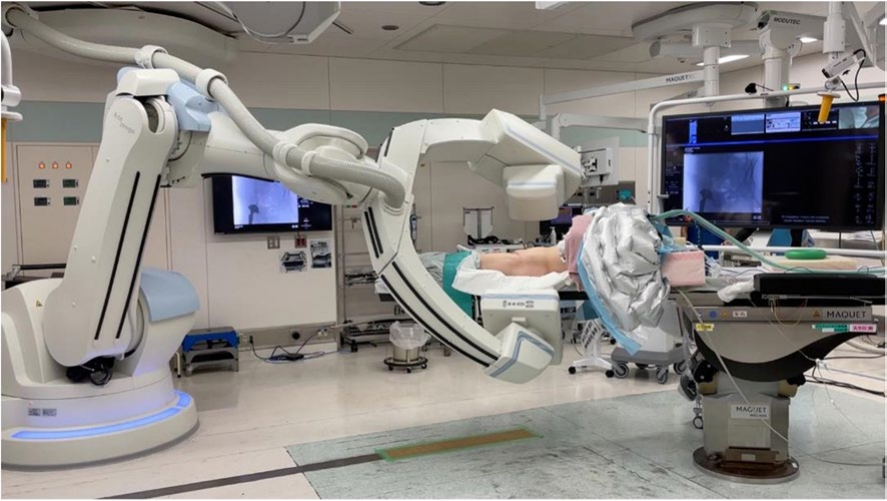
Hybrid operating room equipped with a 3D flat-panel C-arm (Artis Zeego, Siemens). Surgery was performed in the supine position and intraoperative C-arm CBCT was performed under general anesthesia

All operations were performed in a hybrid operating room using a 3D flat-panel C-arm (Artis zeego, Siemens). Surgery was performed in the supine position with mild lift of the affected side and intraoperative C-arm CBCT was performed under general anesthesia (Fig. 1).

- 3D image acquisition technology and planning

**Fig. 2:**
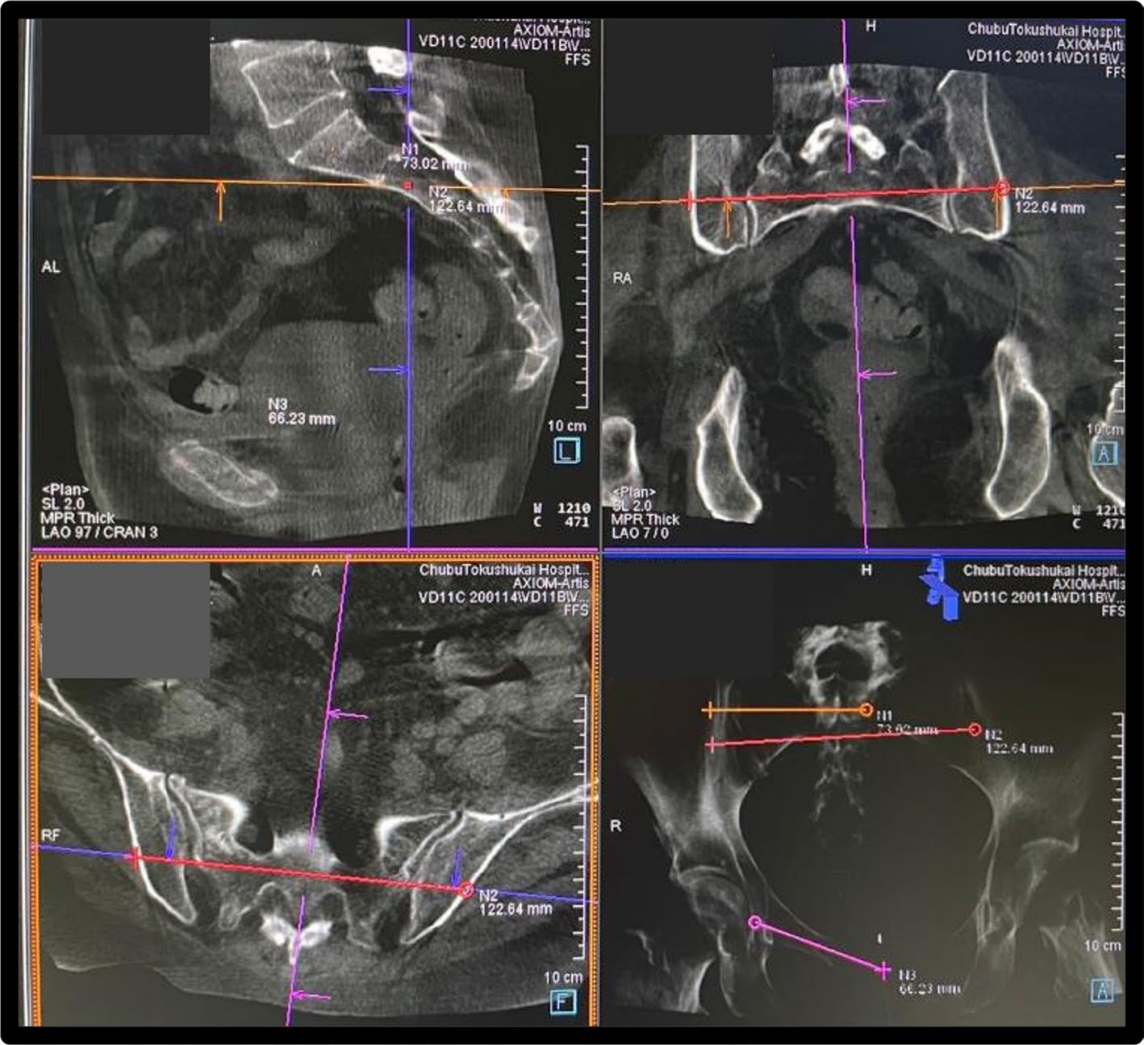
3D preoperative planning. Large field of view and high-quality images were acquired using C-arm CBCT. Data were reconstructed on a workstation. The entry point of the screw trajectory into the cortical bone was indicated using a cross (×) and the end point of the screw tip was indicated with a circle (○).The screw trajectory planning and overlay software used was an inbuilt technology for needle biopsies

After general anesthesia, DynaCT images were acquired for each patient with a 6-s DR program using an angiography flat detector C-arm CBCT (Artis zeego VD11C; Siemens Healthcare, Forchheim, Germany). After image acquisition, the resulting raw projection images were automatically transferred to a post-processing workstation (*syngo* X Workplace; Siemens Healthcare) for 3D volume reconstruction. The reconstructed images had a slice size of 512 × 512 pixels with approximately 391 image slices per volume and a typical size of 0.488 mm^3^ isotropic voxels. The guidewire trajectory planning and overlay software used was equipped for needle biopsy (*syngo* Needle Guidance; Siemens AG). The same workstation was used to plan the guidewire trajectory. The guidewire trajectory through the bone was planned on multiplanar reconstruction images to avoid the perforation of the neural foramen and vascular injury. The IS screw tip was planned to terminate in the middle of the sacrum, the antegrade pubic screw tip was planned to be in the suprapubic branch, the retrograde pubic screw tip was planned to be in front of the acetabulum, and the TITS was planned to penetrate the contralateral iliac cortical bone. The entry point into the cortical bone was indicated by a cross and the end point of the screw tip was indicated by a circle (Fig. 2).

- Surgical technique

All procedures were performed by one of two orthopedic surgeons (M.T. and S.N.). A C-arm CBCT was performed under general anesthesia, the guidewire trajectory was planned on the workstation, and the operation was then performed. The operative site was prepared and draped in a sterile manner. The insertion of the PPSP guidewire was performed as previously reported by Jiao et al. [15]. The virtual trajectory was then projected and overlayed onto the real-time fluoroscopic images and displayed on a dedicated live monitor to use the planned trajectory to align the guidewire in actual 3D space.

**Fig. 3:**
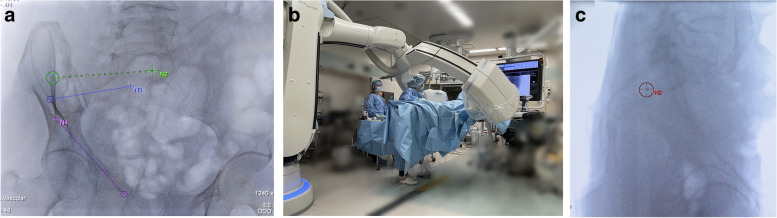
Real-time 3D fluoroscopic navigation. The virtual trajectory was projected and overlayed onto the real-time fluoroscopic images and displayed on a dedicated live monitor (**a**). The C-arm was rotated to the “bull’s eye view” **b** where it was angulated in a way that the cross and the circle displayed on the live monitor completely matched and the central X-ray beam was aligned with the planned trajectory (**c**)

**Fig. 4:**
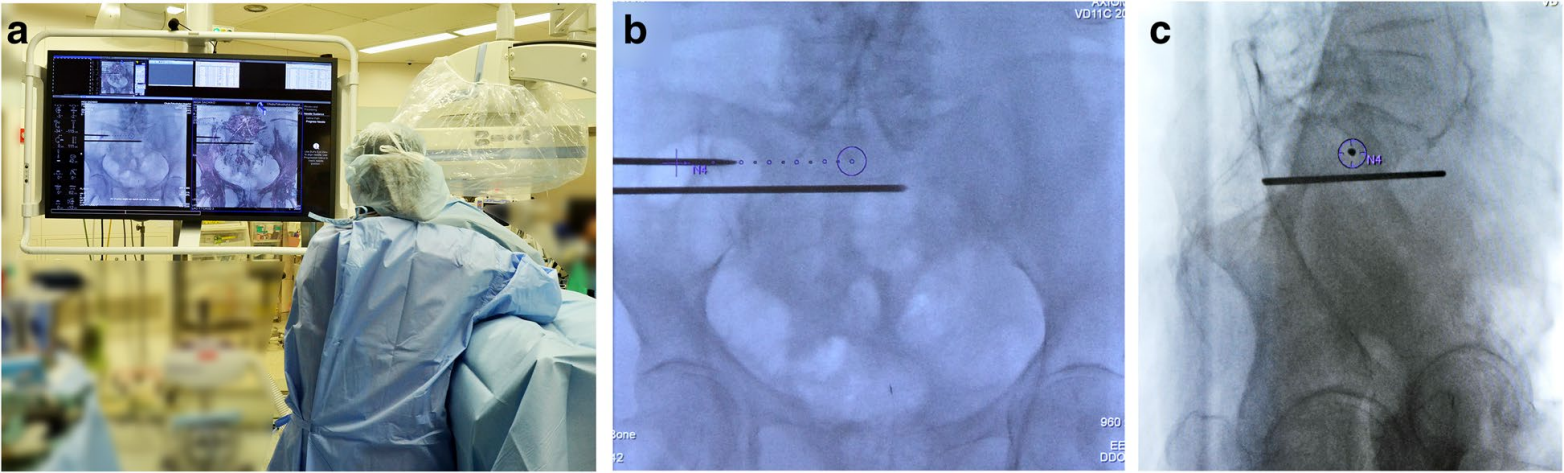
Guidewire insertion using real-time 3D fluoroscopic navigation (**a**). The surgeon is able to insert the guidewire while looking at the real-time image in the AP (**b**) or bull’s eye view (**c**), which is overlaid with the trajectory of the guidewire insertion planned in 3D images. The guidewire is at the center point of the bull’s eye view, with the tip and back end of the guidewire overlapping and located in the center of the circle and cross (**c**)

**Fig. 5:**
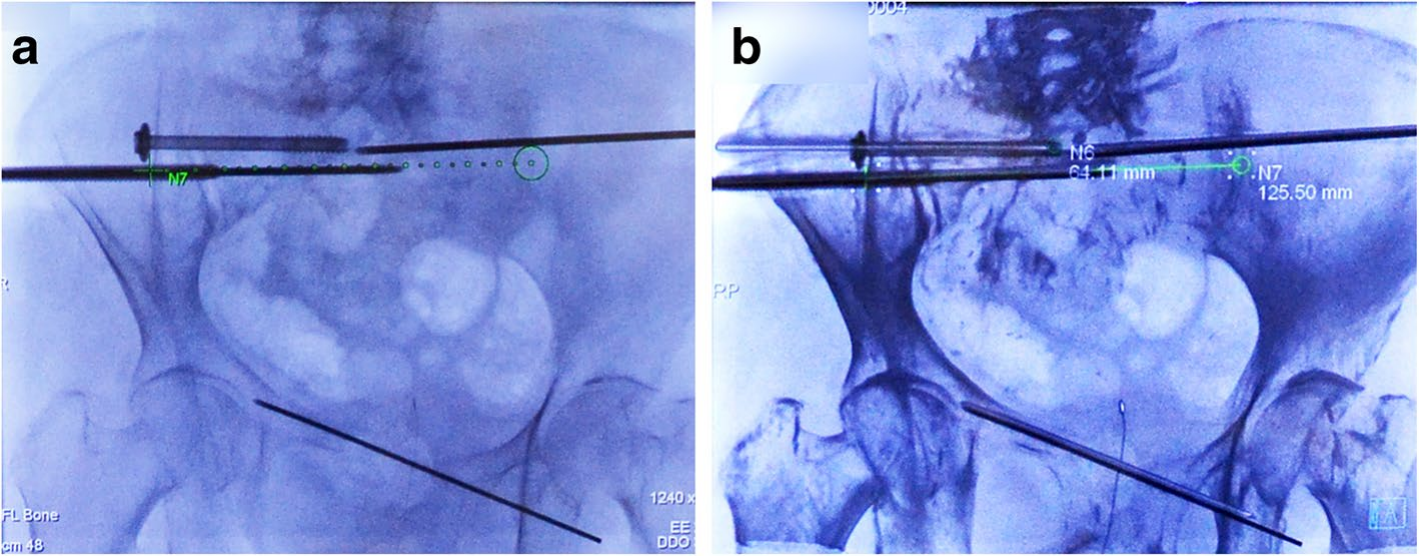
Screw placement under navigation. A 6.5-mm cannulated cancellous screw with a washer was placed under real-time fluoroscopy (**a**) with an overlayed trajectory in 3D preoperative planning (**b**)

The C-arm was rotated to the lateral view (Bull’s eye view) and was angulated so that the cross and circle displayed on the live monitor completely matched; the central X-ray beam was aligned with the planned trajectory (Fig. 3). The skin was then incised 5 mm above the entry point. A 2.8-mm smooth guidewire was then tamped and inserted into the entry point of the cortical bone. The guidewire was inserted by adjusting it so that both the tip and back-end overlapped and were positioned in the center of the circle and crossed so that it was at the center point in the bull’s eye view [15] (Fig. 4). After insertion of the guidewire to more than half of the planned insertion position, C-arm CBCT was performed again to ensure that the guidewire did not perforate the neuroforamina or blood vessels. After confirming that the guidewire was inserted correctly, the distance from the entry to the endpoint was measured on the screen and the screw length was determined. At the time of screw placement, skin incision was extended to 15 mm and a 6.5-mm cannulated cancellous screw (MEIRA Corp, Aichi, Japan) with a washer was placed under real-time fluoroscopy with an overlayed trajectory in the anteroposterior (AP) view (Fig. 5). However, given that it is difficult to insert an antegrade or retrograde pubic screw using a bull’s eye view and also maintain a steep angle against the ilium to drill the entry point of the guidewire, the predrilled of the entry point was performed using this navigation and the guidewire was inserted along the trajectory overlaid on the AP and lateral images using 3D fluoroscopic navigation.

The patient was allowed to transfer to a wheelchair immediately on postoperative day 1, followed by partial weight bearing for 3 weeks and full weight bearing for 6 weeks after the surgery.

**Fig. 6:**
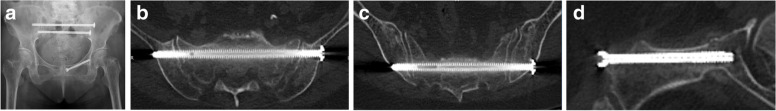
A 79-year-old woman with fragility fractures. TITS screws at S1 and S2 and retrograde pubic screws were placed in postoperative X-ray (**a**). Postoperative CT showed no perforation of the neural foramen, blood vessels, or hip joint (**b**, **c**, **d**)

Clinical and radiographic follow-up was performed immediately after surgery, then at 1, 2, 6, and 12 weeks, and 6 months thereafter. CT was performed 1 week after surgery to monitor hematomas and early straying or backout of the screws (Fig. 6). Clinical evaluation was based on operative time, blood loss, pain at the latest follow-up, gait ability before and after surgery, and the presence of complications. Pain was assessed using the visual analog scale, with < 20 mm classed as pain-free and > 20 mm classed as painful. Gait ability was compared between pre-injury and final observation in the following groups: walking alone, walking with a cane, walking with a walker, in a wheelchair, and bedridden.

Imaging evaluation was also performed by two orthopedic surgeons (M.T. and S.N.) with postoperative CT. Bone healing, screw blackout, and accuracy of the screw placement were investigated. Bone healing was defined as pain relief, the absence of progressive dislocation of the fracture site on X-ray films, or cross-linking or remodeling of the fracture site on CT images after at least 3 months of follow-up. Perforation of the neural foramen or blood vessels was graded according to a classification system for the correct placement of pedicle screws [16] as follows: grade 0, no perforation; grade 1, perforation < 2 mm; grade 2, perforation of 2–4 mm; and grade 3, perforation > 4 mm. Screw misplacement was defined as grade 2 or 3 on postoperative CT. Backout was defined as a screw position that had moved by more than 2 mm compared with the radiograph immediately after surgery.

## Results

Among 41 patients, 121 screws were placed using real-time 3D fluoroscopic navigated screws for FFP. Of these, 29 were IS screws, 49 were TITS screws, five were antegrade pubic screws, 33 were retrograde pubic screws, and five were iliac wing screws (Table 3). There was no misplacement in 121 screws, and grade 1 perforations were found in two cases: one antegrade pubic screw and one retrograde pubic screw. There were no cases of grade 2 or 3 perforations, vascular injuries, perforation to the neural foramen or hip joint, or perforation of the abdominal cavity (Table 3).

**Table 3:** Evaluation of screw perforation [16]

	Grade			
Type of screw (n)	0	1	2	3
IS screw (29)	29	0	0	0
TITS screw (49)	49	0	0	0
Antegrade pubic screw (5)	4	1	0	0
Retrograde pubic screw (33)	32	1	0	0
Iliac screw (5)	5	0	0	0

The mean ± SD operative time was 84 ± 38.7 min, and the mean blood loss was 7.6 ± 3.8 g. The mean time to transfer to a wheelchair after surgery was 2 (1–7) days. Residual pain occurred in 6 of the 41 patients. There were 6 patients with residual pain having a VAS of ≥20 mm; however, none of them had residual knocking pain before the surgery. The VAS of patients with residual pain averaged 28 mm (20–40 mm). The 35 (85%) remaining patients achieved pain relief. Gait ability from preoperative and latest follow-up after surgery was reduced in 11/41 patients (27%) and maintained in 30 patients (73%) (Table 4).

**Table 4:** The change of walking ability

pre-injury (patients)	final observation (patients)
walking alone 21	walking alone 15
	walking with a cane 2
	walking with a walker 3
	in a wheelchair 1
walking with a cane 15	walking alone 1
	walking with a cane 10
	walking with a walker 3
	in a wheelchair 1
walking with a walker 5	walking with a walker 4
	in a wheelchair 1

There was no proceeding anemia, neurological deficit, or deep infection during the follow-up period.

Bone union was achieved in all 41 patients at the latest follow-up (Table 5). Screw blackout was identified in 15% (18/121 screws). Blackout of the screws showed no progression after bone union was achieved. There was no removal of the implants as they did not irritate around the screws. The radiation duration averaged 12.5 minutes (3.7–34.5 minutes).

**Table 5:** Clinical outcomes

Characteristics	Values or proportions
Operative time (min)	84 (34–210)
Blood loss (g)	7.6 (3–20)
Days to transfer to a wheelchair (days)	2 (1–7)
Pain remains (%)	15
Walking ability	
Maintained (%)	73
Reduced (%)	27
Bone healing (%)	100

## Discussion

The present study showed that PPSP with real-time 3D fluoroscopic navigation for FFP in a hybrid operating room was effective and enabled accurate screw placement with minimum complications, early mobilization, and improved pain relief. The application of an already installed needle biopsy application to PPSP navigation is novel.

Richter et al. reported that the advantages of the hybrid operating room included improved image quality and enlargement of the display window, which increased the accuracy of computer-assisted IS screws [17]. To our knowledge, the use of C-arm CBCT in hybrid operating rooms is an advanced 3D imaging technology. Cross-sectional image information can be overlayed and integrated with real-time fluoroscopic images. The resulting image information can be used in various fields, such as needle biopsy of malignant tumors, cardiovascular surgery, neurosurgery and spine surgery, using existing 3D fluoroscopic navigation techniques for needle guidance [15, 18–21]. In the present study, this accurate and precise technique was applied to guidewire insertion of PPSPs in pelvic fractures.

Hybrid operating rooms allow the use of 3D fluoroscopic navigation because of the link between the X-ray radiolucent operating table and data acquired by the C-arm CBCT. Therefore, there is no need for a dedicated navigation system or placement of a tracker on the patient’s iliac bone. The advantage of this system is that the surgeon can insert the guidewire while looking in real-time at the fluoroscopic image, which is overlayed with the trajectory of the screw planned in 3D images. This intraoperative procedure can be used to determine the direction and length of the screw while checking its trajectory. Moving the angle of the C-arm so that the entry and endpoints are in a straight line allows the bull’s eye view to be used to navigate to the position of the skin incision, resulting in a small skin incision. The guidewire is inserted while looking at the bull’s eye view and the entry point and direction of insertion are navigated simultaneously to eliminate the need for extra deployment and avoid damage to the surrounding soft tissue or blood vessels. In this procedure, intraoperative CT images were acquired twice to confirm the direction of the guidewire and allow us to determine the screw length for secure fixation. This procedure may provide good clinical results, minimize complications, improve pain relief and assist bone healing. In the present study, wheelchair transfers were allowed the day after surgery, and a 3-week non-weight-bearing period was ensured as the patients had FFP. Given that bone healing was achieved in all patients, an early postoperative weight-bearing period might be considered.

One of the disadvantages of PPSP is the requirement for a high radiation dose in the hybrid operating room due to the use of multiple intraoperative C-arm CBCT scans. PPSP is a safe technique, especially for patients with challenging sacral anatomy and a narrow corridor. In such patients, at least three intraoperative CT scans might be necessary to reduce the incidence of complications owing to screw misalignment. We made every effort to reduce the radiation dose to the patient by using lower CT imaging doses. The radiation dose to the surgeon was reduced by maintaining a distance of at least 2 m and waiting behind a protective board during the C-arm CBCT scans [22]. The risks and benefits to the patient should be considered with regard to radiation exposure, to ensure that the PPSPs can be inserted safely and reliably. In a future study, we intend to compare hybrid operating room exposure with other imaging modalities.

In addition, all the patients underwent CT during the first postoperative week. Changes in postoperative vital signs or hematomas were not observed in any of the patients, and bone healing was achieved in all the patients; therefore, CT evaluation during the first postoperative week should be repeated to reduce radiation exposure.

The navigation in the hybrid operating room is registered to the patient’s bed and any change in patient position after CT acquisition leads to errors in the navigated guidewire trajectory. The system’s method for correcting for errors involves the ability to perfectly overlay real-time images and intraoperative 3D data to maintain accuracy. On the other hand, it may be difficult to perform PPSP in the lateral decubitus position, which does not allow a secure patient position, and supine or prone positions may be preferred for this procedure. Therefore, PPSP for FFP using a real-time navigation system in a hybrid operating room requires sufficient knowledge to make full use of this advanced technology due to the narrow safety zone for screw placement.

This study has several limitations. First, this study was a retrospective case series. The outcomes of surgical treatment and conservative treatment should be compared. Second, it demonstrated the capabilities of intraoperative C-arm CBCT imaging in a hybrid operating room and also improved the accuracy of PPSP in real-time navigation by interfacing with existing applications. Future studies are required to compare our system with that of 3D navigation, which is less exposed to radiation, but considering the invasiveness of placing the tracker in the pelvis, the accuracy of registration, and the fact that the trajectory cannot be overlayed on the real-time image.

## Conclusion

The present study examined 41 patients with FFP treated using PPSP in a hybrid operating room with real-time 3D fluoroscopic navigation. Surgery time was not short, but the blood loss was minimal, and all patients were able to transfer from the bed after surgery. A total of 121 screws were placed and bone healing was achieved in all cases, with minimal complications. Therefore, use of this 3D fluoroscopic navigation system in the hybrid operating room is useful for PPSP for FFP without necessary of an additional navigational system.

## Supplementary Information

**Additional file 1.**

## Data Availability

All data used and analyzed during the current study are available from the corresponding author on reasonable request.

## Abbreviations

FFP: Fragility fractures of the pelvis
PPSP: Percutaneous pelvic screw placement
IS: Iliosacral
TITS: Transiliac transsacral
CBCT: Cone-beam CT
3D: Three-dimensional
AP: Anteroposterior
